# Designing for Wayfinding in VR: Linking Navigation Interfaces to Spatial Learning and Cognitive Mapping

**DOI:** 10.1145/3772318.3791145

**Published:** 2026-04-13

**Authors:** Armin Mostafavi, Zhiwen Qiu, Tong Bill Xu, Wenqian Niu, Saleh Kalantari

**Affiliations:** Department of Human Centered Design, Cornell University, Ithaca, New York, USA; Information Science, Cornell University, Ithaca, New York, USA; Department of Human Centered Design, Cornell University, Ithaca, New York, USA; Department of Human Centered Design, Cornell University, Ithaca, New York, USA; Department of Human Centered Design, Cornell University, Ithaca, New York, USA

**Keywords:** VR Wayfinding, spatial learning, navigational performance, locomotion, teleportation, viewpoint transition

## Abstract

Various virtual locomotion techniques and visual transition methods are used in VR-based navigation research, yet few studies have systematically examined their effects on spatial learning, cognitive map formation, and navigational performance in complex indoor environments. We conducted a between-subjects study (N=142) in two high-fidelity VR hospital contexts, including free exploration and task-based wayfinding, while treating locomotion and viewpoint transitions as experimental factors. Spatial learning was measured through pointing, distance estimation, and sketch-map accuracy; performance was measured through completion time and distance traveled; and experience was measured through cybersickness, perceived presence, and usability. Locomotion techniques affected task completion time, with teleportation associated with faster performance in the task-based context. Spatial learning effects were mixed, with patterns indicating that techniques without viewpoint transitions may better support cognitive mapping. Empirical insights and guidelines are provided to improve the reliability and real-world applicability of VR-based wayfinding research.

## 1 Introduction

Virtual reality (VR) utilizes advanced head-mounted displays (HMDs) and interactive three-dimensional (3D) user interfaces that enable users to manipulate objects and navigate within virtual environments [6, 62]. The virtual locomotion technique (VLT) is a fundamental component of VR systems that translates physical body movements (e.g., gestures or head-rotation) and inputs from digital devices (e.g., joysticks or treadmills) into virtual orientation, motion, and viewpoint transitions [8, 46, 86]. The design of VLTs can contribute to experiences of discomfort, disorientation, or nausea while performing tasks such as wayfinding, searching for objects, or inspecting scenes in VR [62, 72, 89]. Therefore, evaluating the effectiveness of VLTs across various levels of interactions is essential to improving user experience in virtual spaces.

Researchers have demonstrated that different forms of VLTs can influence simulator sickness [31, 97], navigational abilities [11, 90, 96], and spatial cognitive abilities [5, 9, 28]. In general, locomotion techniques that support natural physical forms of interaction, such as translating real-world walking motions on a tread-mill into virtual movement, have been found to be more beneficial for effective navigation in VR [26, 106, 121]. Similarly, controller-based steering methods, which enable users to control the direction and speed of continuous virtual motion, provide an uninterrupted optical flow that emulates aspects of real-world locomotion, thereby supporting spatial understanding and path integration [12, 13, 114]. However, such forms of motion have been associated with greater cybersickness, most likely due to sensory conflicts as the user’s vestibular system processes visual cues without corresponding physical sensations [57, 59]. Additionally, navigating large-scale interior environments such as hospitals or transportation hubs with realistic VLTs requires considerable time and physical effort, and users may become impatient with the need to traverse extended distances. In contrast, another locomotion technique that is frequently used in VR applications is teleportation, in which users select a destination and are instantly moved to that point. This type of VLT tends to reduce cybersickness by eliminating optical flow or acceleration cues, but the abrupt view changes may increase disorientation and negatively impact immersion and spatial awareness of the environment [18, 19, 90].

There is a growing trend in recently developed VLTs that seek to balance the benefits and drawbacks of teleportation and continuous movement [84], for example by integrating viewpoint transition scenes when teleporting, or by using a mixture of both strategies [5, 16, 86]. Some researchers have applied a joystick-based discrete rotational method that updates the user’s orientation at fixed intervals, instead of continuously, and have found this effective for cybersickness reduction [15, 124]. Yet another approach is to implement visual interventions such field-of-view (FOV) restrictions, peripheral blurring effects, and dynamic depth-of-field (DOF) blur to obscure distant non-focal cues during continuous locomotion [25, 36, 71]: VLT approaches that lean more toward teleportation may benefit from incorporating rotational self-motion cues, orientation indicators at landing points, and smooth viewpoint transitions to improve orientation and spatial knowledge acquisition [40, 67]. Some studies have investigated the “HyperJump” approach, which seamlessly combines continuous and teleportation techniques by interspersing steady movement with regular-interval jumps to reach the destination more quickly. This approach may be particularly beneficial for balancing spatial orientation with usability in large-scale virtual settings [5].

Despite the growing diversity of virtual locomotion techniques and visual transition methods in VR-based navigation, few studies have systematically examined how these design choices influence spatial learning, cognitive map formation, and navigational performance in complex, realistic environments. Most prior work has focused on outdoor or abstract virtual spaces, or has isolated outcomes such as cybersickness or task time, often overlooking the cognitive implications of movement mechanics on internal spatial representations. This presents a critical gap, as environments such as hospitals, airports, and educational institutions require users not only to reach destinations quickly but also to build accurate spatial knowledge, essential for tasks like returning to key locations, orienting to signage and landmarks, and supporting safety-critical operations. Beyond these applied domains, VR navigation is increasingly used in cognitive training and rehabilitation [7, 120], where locomotion design directly influences the effectiveness of interventions aimed at strengthening memory, orientation, and executive function.

To address this gap, we developed a high-fidelity VR platform simulating realistic hospital settings and conducted a large-scale controlled experiment (N = 142) evaluating four locomotion approaches that combine continuous and discrete movement with visual transition techniques (blink, tunneling). Our study goes beyond prior work by:

- Systematically comparing multiple commonly used locomotion and transition techniques within one unified, ecologically valid platform.
- Employing a two-phase design (free exploration vs. time-pressured wayfinding) to examine how locomotion techniques influence both wayfinding performance and configurational learning under different task demands.
- Integrating multiple outcome measures, behavioral performance, spatial-learning assessments (pointing, estimation, sketch maps), and user-experience factors (cybersickness, presence, usability), to capture the full spectrum of trade-offs.
- Situating locomotion studies in ecologically valid environments, thereby establishing design guidelines for selecting and configuring movement and viewpoint transition techniques when developing VR-based navigation systems for applications such as human–environment interaction studies and cognitive training and rehabilitation interventions, among others.

## 2 Related Work

### 2.1 VR for Wayfinding, Spatial Learning, and Cognitive Mapping

Multiple studies report a significant correlation between VR and real-world wayfinding performance, supporting the ecological validity of VR for navigation research. This holds for both young and older adults, though the strength of the correlation can depend on task difficulty and population [32, 41, 49, 73]. VR-based wayfinding tasks can predict real-world navigation ability, especially as task complexity increases [29, 41]. In immersive VR, wayfinding performance (success in reaching goals) is largely comparable to real environments, but visual attention and information processing differ: participants process visual information more efficiently in real settings, while VR users search more efficiently [50].

Studies directly comparing distance covered and time spent in the VR environment show mixed results. Some report that tasks are completed faster in VR, possibly due to differences in movement mechanics (e.g., “gliding” in VR vs. walking in reality), but this can make VR distances feel less effortful and less realistic[33, 35]. In some cases, participants in VR cover similar or slightly longer distances due to less efficient route choices or increased exploration, especially in complex or multi-level environments[35, 49, 58, 107]. VR may not fully replicate real-world sensory cues (e.g., physical effort, multisensory input), which can affect navigation strategies and perceived workload[33, 73, 107]. Differences are more pronounced in complex, multi-level, or unfamiliar environments [35, 58, 107]. VR is a valid tool for studying wayfinding, but results should be interpreted with awareness of these differences.

The way users move in VR, through continuous locomotion, joystick steering, teleportation, or hybrid techniques, has a significant impact on distance traveled, time spent, spatial learning, and cognitive map formation (see 2.2). Continuous methods, such as joystick control and redirected walking, better approximate real-world navigation and generally support accurate distance estimation and path integration [23, 37, 51, 60]. Teleportation, by contrast, enables rapid movement and reduces cybersickness [39] but can cause spatial disorientation, path integration errors, and weaker spatial memory compared to continuous steering methods[19, 53, 85]. Mitigation strategies like visualized trajectories and traceable teleportation reduce disorientation and partially restore spatial awareness [20, 40, 47]. Hybrid techniques, such as HyperJump, aim to balance efficiency with orientation preservation, while redirected walking and other natural methods provide the strongest cognitive map accuracy, albeit with practical limitations in small VR setups [27, 102].

The impact of these movement techniques on spatial learning and cognitive map formation is not yet well understood. For instance, some studies have found that teleportation can hinder the development of accurate cognitive maps, especially for distance and route knowledge, though enhancements like visualized paths or undo-redo features have been found to mitigate some deficits [47, 91]. Controller-based movement (joystick, grab, slider) seems to offer a middle ground, with continuous movement supporting spatial awareness better than teleportation, but often less so than physical walking [5, 23, 91]. In case of redirected walking and other natural or semi-natural techniques, there is evidence that they may support better spatial learning and cognitive map accuracy, as they provide rich sensory and proprioceptive feedback [27, 38, 102]. However, only a limited number of studies have been published on these topics, and some findings have been inconsistent.

Overall, the existing literature suggests that locomotion techniques can differentially shape how users perceive and learn virtual environments, but the evidence remains mixed and often context-dependent. Teleportation, while efficient and low in physical demand, has been linked to weaker spatial learning, though design enhancements can mitigate some of these drawbacks. Continuous controller-based methods offer a balance between usability and spatial awareness, while more natural approaches, such as redirected walking, may provide the strongest support for cognitive mapping, albeit with practical limitations. Given these trade-offs, the choice of locomotion technique should be aligned with the goals of a given application: for example, naturalistic methods may be most appropriate for spatial training, whereas teleportation may be more suitable for rapid exploration. Importantly, because prior studies vary in methodology and often focus on open or abstract spaces, there is still a limited and sometimes inconsistent understanding of how locomotion methods affect spatial learning in complex, realistic indoor settings. This gap motivates the present research, which systematically investigates how different locomotion and transition techniques influence navigation performance, spatial learning, and cognitive map formation in high-fidelity VR environments.

### 2.2 Controller-based Travel Techniques

Researchers have explored a wide array of controller-based travel techniques in VR [31, 66], including walking-based, steering-based, and selection-based methods [6]. While walking-based navigation closely resembles natural movement and rotation in the physical world, providing high interaction fidelity [74] and notable benefits in spatial orientation and feelings of presence [92, 95, 103], the practical constraints of workspace sizes and sensor-tracking ranges often restrict its use. Steering-based methods enable greater ease when traveling across extended virtual distances by allowing continuous directional control through physical inputs (i.e., controlled by steering devices) or gestures (i.e., controlled by gaze, hand, or body learning) without the need for actual walking [62]. For example, Sarupuri and colleagues [98] developed TriggerWalking, a technique that simulates bipedal locomotion by allowing users to alternate between the trigger buttons of one or both controllers while adjusting speed by changing the angles of the controllers. They evaluated TriggerWalking and found it was easy to learn, less physically demanding than walking-in-place, and induced less cybersickness compared to joystick locomotion. Kitson and colleagues [55] evaluated joystick-based control, head-directed steering, and three chair-based directional leaning interfaces. Their results revealed that joystick-steering was favored for comfort and precision, and provided better spatial orientation, accuracy, and ease-of-use, despite issues with controllability and stability. Xu and colleagues [119] compared joystick and walking-in-place in a spatial knowledge acquisition task; these researchers found that both techniques resulted in a comparable average object placement error, indicating similar levels of spatial memory accuracy. Controller-based techniques offer a range of approaches to balance user comfort, spatial orientation, and task performance in virtual environments.

Teleportation is a common controller-based interface that involves discrete movement by automatically shifting the viewpoint to a selected target destination [62]. Teleportation does not produce any optical flow during discrete jumps, thereby lowering the risk of cybersickness and offering higher usability and enjoyment compared to continuous steering-based navigation [39, 70, 109]. However, early research by Bowman and colleagues [19] indicated that the abrupt view changes in teleportation led to spatial disorientation during travel. Paris and colleagues [85] analyzed continuous and discrete locomotion techniques to determine their effectiveness in transmitting self-motion cues in an immersive virtual game environment; they did not find significant differences in simulator sickness or perceived presence, but their results indicated that teleportation tended to result in more path integration errors and spatial disorientation. Studies by Buttussi and Chittaro [22] and Kim and colleagues [53] also found that teleportation can negatively affect spatial understanding, resulting in poorer performance in spatial memory tasks compared to joystick-steering methods. Efforts to minimize disorientation in teleport-based VLTs generally focus on visualization technologies that display linear or curved trajectories from the current location to the destination prior to movement activation [20, 40].

### 2.3 View Transition Techniques and Spatial Knowledge Acquisition

Several systematic taxonomy studies have characterized elements of 3D-travel techniques, including input conditions, direction/path control, rotation, and acceleration/velocity [19, 80, 104]. Prior researchers have emphasized how the interplay among such factors can lead to trade-offs between spatial knowledge acquisition and cybersickness [10, 89]. In one notable study, Bowman and colleagues [18] investigated the impact of four widely different teleportation speeds on spatial understanding during an object-locating task, and found no significant differences in spatial awareness across the different levels of velocity, indicating that this factor may not be very important to the impacts of teleportation. Bolte and colleagues [17] introduced the concept of “Jumper” VLTs, which allow users to walk naturally for short distances and make virtual jumps for longer distances, with smooth viewpoint transition animations. Results from a map-sketching task revealed that the Jumper technique only slightly diminished spatial memory performance compared to real-world walking, and was favored by users over standard teleportation for its less disruptive transitions. Weissker and colleagues [114] compared the spatial awareness between continuous steering and jumping for repeated teleportation and discovered that both methods achieved similar spatial updating accuracy, while jumping caused much less simulator sickness.

Bhandari and colleagues [16] proposed the “Dash” VLT approach, which incorporates a small amount of optical-flow cues to enhance path integration and maintain orientation during teleportation. A subsequent study by Rahimi and colleagues [90] evaluated three automated viewpoint transition techniques, instant teleportation, animated interpolation, and pulsed interpolation with sequential intermediate views, on spatial awareness during outdoor scene transitions. They showed that animated interpolation allowed for the best spatial awareness in terms of object-tracking tasks, but that it also induced more simulator sickness compared to the other methods. Building on these findings, Adhikari [5] developed the “HyperJump” technique, which was intended to seamlessly merge continuous short-distance travel with fast-paced jumps to facilitate more rapid navigation without compromising spatial memory.

Other studies have focused on viewpoint rotations rather than changes of position. Riecke and colleagues [93] and Klatzky and colleagues [56] demonstrated that participants could better maintain spatial updating during rotations if the VLT displayed landmark-rich visual motion cues. Cherep and colleagues [28] similarly emphasized the importance of rotational self-motion cues in spatial cognition, showing that a partially concordant teleportation interface (i.e., using a controller to translate but physically turning the body to rotate) resulted in fewer errors compared to a discordant interface (i.e., using a controller to both translate and rotate). This was further confirmed by Lim and colleagues [67], who found that the partially concordant method could significantly improve spatial knowledge acquisition in object-to-object pointing and sketch-map tasks. Zielasko and colleagues [124] systematically evaluated virtual rotation techniques and discovered that joystick-controlled methods excelled in search-task completion times, while joystick and body-based rotations showed similar performance in spatial learning for pointing and configurational tasks.

In summary, previous research suggests that the type of visual cues and motion-control used in VLTs may have a strong effect on spatial learning. However, this body of literature is limited and unsystematic (with different studies focused on different sets of VLTs and different outcome measures), and the vast majority of the research has been conducted in open outdoor virtual settings, where large distances are the norm and where spatial knowledge acquisition may develop differently compared to complex indoor environments.

### 2.4 Cybersickness and Spatial Learning

Cybersickness, often experienced during VR-based navigation, encompasses symptoms such as headache, eye strain, and nausea [61] and typically arises from a sensory conflict between visual and vestibular signals, such as perceiving motion visually while being physically stationary. Its severity can be influenced by intrinsic factors (e.g., age, gender) and technical parameters such as optical flow, motion intensity, and head-mounted display ergonomics [30, 57, 64]. A wide range of mitigation strategies has been explored to improve user comfort and enable longer engagement in virtual environments. Among these, field-of-view (FOV) restriction techniques have received significant attention for their effectiveness in reducing simulator sickness by narrowing peripheral vision during movement or rotation [54, 68, 115].

For example, Fernandes and Feiner [36] developed a dynamic FOV restrictor that subtly reduced the visible field when motion began and gradually restored it upon stopping, enabling participants to spend more time navigating VR environments with minimal awareness of the intervention. Similarly, Zhang et al. [87] introduced “tunnel vision” smart glasses using switchable polymer dispersed liquid crystal (PDLC) film to dynamically adjust peripheral visibility according to motion intensity, while Carnegie and Rhee [25] proposed a gaze-contingent depth-of-field (DOF) approach that adapts blur levels based on the viewer’s fixation depth to reduce perceptual conflict during focus transitions. Nie et al. [81] further extended this approach by implementing a real-time saliency-based blurring technique, adjusting peripheral vision clarity depending on the task relevance of visual elements. Other techniques include snap-rotating viewpoints to minimize motion discontinuities [34], manipulating static blur windows [69], and employing asymmetric FOV configurations to enhance comfort during locomotion [4, 118].

While these methods effectively alleviate cybersickness, they introduce potential trade-offs for spatial learning, cognitive mapping, and task performance. Restricting peripheral cues, for instance, may reduce access to critical visual information needed for forming accurate mental representations of space. Barhorst-Cates et al. [12] demonstrated that severe FOV limitations (≤ 4°) significantly impair spatial memory and require participants to allocate greater cognitive resources during a landmark pointing task, highlighting the cost of overly aggressive sickness mitigation strategies. Similarly, saliency-driven and blur-based techniques [25, 81] stabilize motion perception but may inadvertently limit environmental awareness and hinder the development of robust cognitive maps. These findings are particularly relevant for VR-based wayfinding studies, where accurate spatial learning and orientation are central research goals.

Presence, defined as the subjective sense of “being there” in a virtual environment, has emerged as a crucial mediator between cybersickness and spatial cognition. Cybersickness has been shown to negatively correlate with presence [100, 112], suggesting that discomfort can disrupt embodied engagement and reduce the quality of spatial encoding. However, results are not uniform: Lin et al. [69] reported that dynamic FOV adjustments can reduce cybersickness without diminishing presence, while Teixeira and Palmisano [48] found that presence is positively associated with vection (the illusion of self-motion) rather than sickness levels. Since vection contributes to embodied navigation, these findings imply that preserving immersive qualities supports spatial orientation and cognitive map formation even when sickness mitigation techniques are used.

Table 1 shows an overview of existing high-quality studies examining spatial learning and cognitive mapping as related to VR locomotion and view-transition techniques. Collectively, this body of work reveals the importance of designing balanced VR navigation interfaces. Locomotion techniques, viewpoint transitions, and cybersickness mitigation strategies must be carefully coordinated to minimize discomfort while preserving spatial learning fidelity. Given the growing diversity of continuous and discrete locomotion techniques and visual transition methods, adopting a comparative approach is essential to understand how these design choices influence time spent in VR, spatial cognition, task performance, and presence. Such insights are critical for developing evidence-based guidelines for VR-based navigation research and ensuring the reliability of studies examining cognitive mapping, distance estimation, and wayfinding performance.

A persistent challenge in this domain is the lack of commensurability across VR navigation studies. Findings are often difficult to compare because experiments employ different locomotion and transition techniques, which can substantially influence navigation strategies, spatial learning, and cognitive map formation [5, 60]. Even small variations in interface design, such as continuous steering versus teleportation, can lead to inconsistencies in measured outcomes, making it difficult to synthesize results across studies. This highlights the need for systematic evaluations of movement and viewpoint transition techniques to improve methodological consistency and support more meaningful comparisons within VR-based wayfinding research.

Another concern relates to the ecological validity of VR findings, particularly when studies rely on non-natural locomotion techniques such as teleportation. Research in environmental psychology and spatial cognition has shown that discontinuous movements may produce spatial learning outcomes that do not generalize well to real-world navigation, where proprioceptive and vestibular cues play an essential role in orientation and memory formation [27, 47, 96]. These methodological considerations are especially critical in applied domains, such as healthcare design, rehabilitation, and aging research, where VR-based navigation tasks are increasingly used to train spatial skills or assess cognitive function [111, 120]. By addressing these challenges, the current study responds to a critical need for systematic comparisons of locomotion and viewpoint transition techniques in high-fidelity, ecologically valid environments, enabling more reliable interpretations and applications of VR-based navigation research.

## 3 Virtual Translation And Transition Technique Design

Different VR and gaming industries support a variety of VLTs for both developers and players. For example, Oculus, now part of Meta, supports Teleport Mode and Slide Mode (similar to CS) in Meta Horizon games [75], and offers techniques such as “Blink” and “Snap Rotation” for developers creating new environments using game engines [76]. Similarly, Google VR supports Teleportation [2], Tunneling [3], and Chase Camera [1] techniques in its VR games. Current game engines, such as Unreal Engine and Unity, enable developers to adopt existing industry-standard locomotion techniques, make adjustments, or create custom solutions from scratch. However, the impact of different VLTs on spatial learning, presence, and cybersickness remains unclear. Therefore, this study compared the most frequently used techniques and investigated their effects.

Natural locomotion methods such as real walking and walking-in-place (WIP) were excluded due to feasibility and methodological constraints. Real walking in hospital-scale environments requires paths up to 80 m or more, which is impractical in typical lab spaces without redirected-walking infrastructure or omnidirectional treadmills [82, 83]. WIP, although more space-efficient, has been shown to degrade spatial awareness compared to real walking and introduces high intra-participant variability in stride amplitude, rhythm, and fatigue, leading to inconsistent movement speed and exposure duration across participants and conditions [42, 117, 123]. In contrast, controller-based continuous locomotion and teleportation enable standardized movement speed, consistent exposure time, and uniform optic-flow across participants and conditions, which is crucial for isolating the effects of locomotion-interface design on spatial learning while minimizing confounding differences due to physical motion style [91, 94].

**Figure 1:**
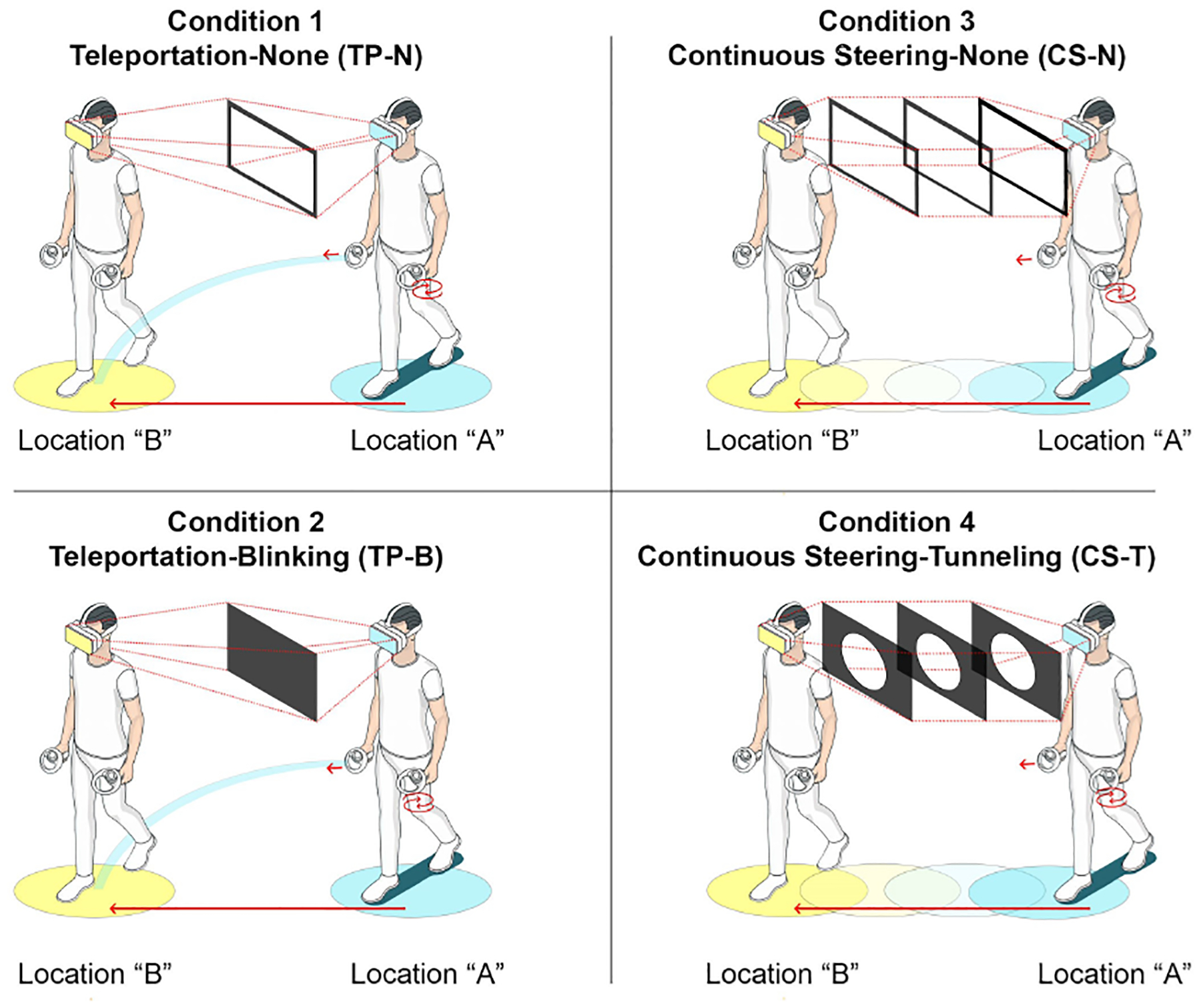
Overview of the four virtual locomotion techniques evaluated in the study: Condition 1, Teleportation with no transition effect (TP-N); Condition 2, Teleportation with Blinking effect (TP-B); Condition 3, Continuous Steering with no effect (CS-N); and Condition 4, Continuous Steering with Tunneling effect (CS-T).

Figure 1 illustrates the four locomotion techniques assessed in the study, featuring two movement types (Teleportation vs. Continuous Steering) and two transition effects (Blinking vs. Tunneling/Restricted FOV). In all conditions, participants could rotate their view either by physically turning in place, which provided a smooth view rotation and change in movement direction, or by using the left joystick on the controller, which enabled temporary 45-degree rotational view changes without altering the direction of movement. The controller option for view rotation was provided to help participants minimize unintended directional drift when looking around, an issue that was identified in our pilot research. When users physically rotate their bodies, they often instinctively start stepping forward and drift away from their intended path or initial heading, which can lead to unintended movement outside the target trajectory, whereas controller-based rotation enables re-orientation without inducing accidental translation. In practice, participants quickly learned to use this controller setup, and we observed minimal problems with unintentional drift during the experiment. This approach aligns with prior findings in locomotion research indicating that controlled rotational input can reduce drift and improve participant comfort [34, 61, 124].

In the Teleportation (TP) conditions (Conditions 1 and 2), participants used the forward joystick button on the right controller to point to their desired location within a 3-meter range. Movement was only possible if the teleportation ray was unobstructed by obstacles, such as walls, barriers, or other non-navigable areas. For transition effects, Condition 1 had no transition effect during teleportation, while in Condition 2, participants encountered a brief blink effect, simulating a blink (fade-to-black) transition during each teleportation event.

**Figure 2:**
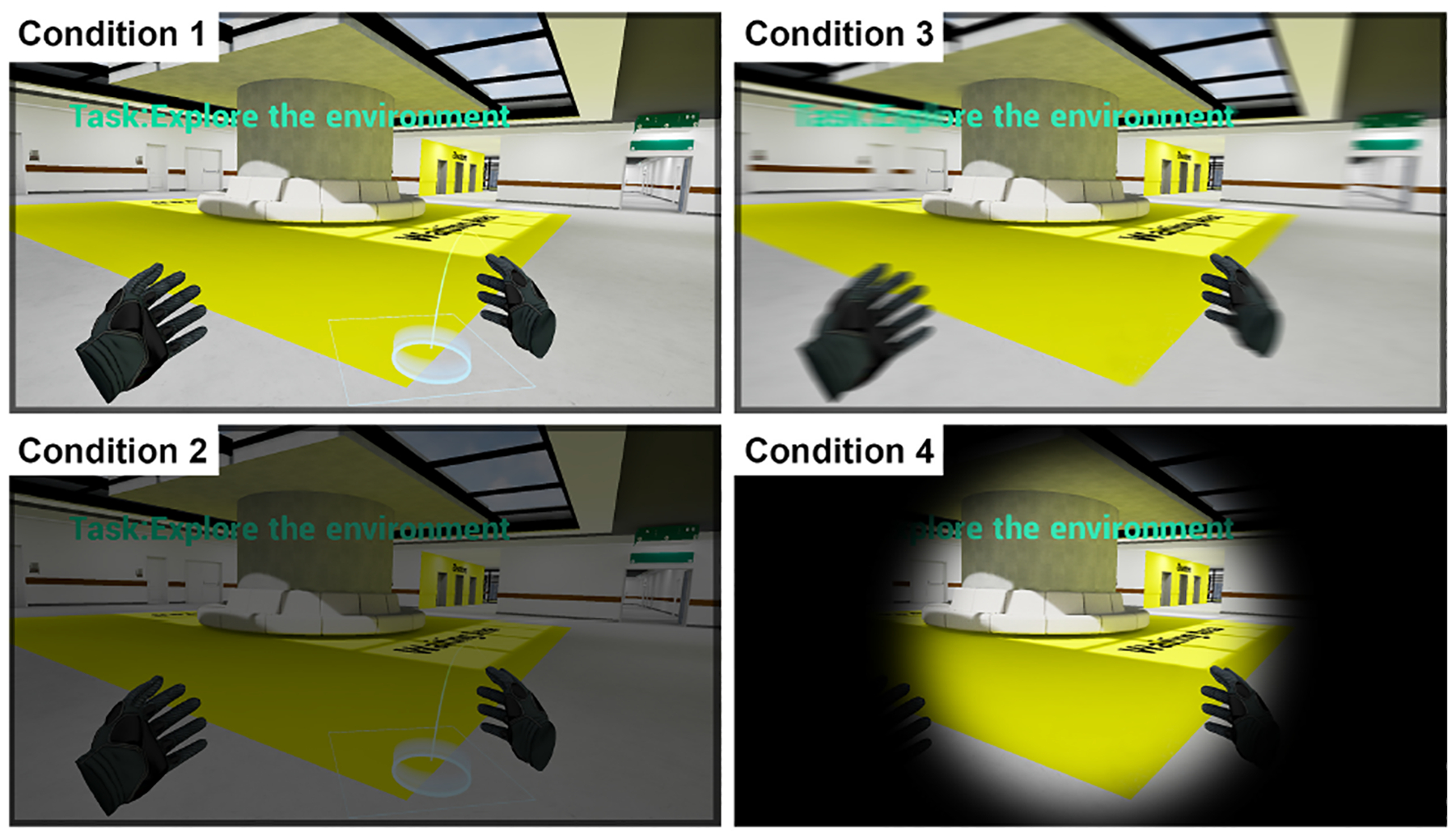
Screenshots of the four VR locomotion techniques (VLTs) during navigation in the Exploration level: Condition 1 (TP-N), Teleportation with no visual transition; Condition 2 (TP-B), Teleportation with Blink (fade-to-black) transition; Condition 3 (CS-N), Continuous Steering with head-aligned forward movement and no visual transition; Condition 4 (CS-T), Continuous Steering with Tunneling effect (restricted field of view) applied during forward motion.

In the Continuous Steering (CS) conditions (Conditions 3 and 4), participants moved forward at a constant speed (1.3 m/s) by pressing the forward joystick button on the right controller, provided the path was free of obstructions. The direction of movement was aligned with the participant’s head orientation. Participants could momentarily stop movement by releasing the right-hand joystick, and the option for left-hand joystick-based rotation allowed them to look around without the need for physical rotation, thereby mitigating unintended directional drift. This approach utilized a push-and-release mechanism, allowing participants to start and stop motion and view changes seamlessly. For transition effects, Condition 3 had no transition effect during movement, while in Condition 4, a Restricted Field of View, or “tunneling effect,” was implemented for the entire duration of forward movement, reducing visual motion cues to enhance user comfort and mitigate cybersickness symptoms. Figures 1 and 2 show comparisons of the four VLT conditions.

## 4 User Study

After developing prototypes of the four different VLTs, we conducted an experiment to evaluate their effectiveness on spatial learning, wayfinding performance, cybersickness, presence, and usability. The experiment employed a between-group design to systematically understand the impacts within a simulated complex indoor environment.

### 4.1 Participants

Prior to any research activities, the study procedures were approved by the Institutional Review Board at Cornell University. A total of 142 participants with diverse demographic backgrounds were recruited using a targeted convenience sampling method, including word-of-mouth and announcements on university e-mail lists. All participants were informed about the objectives and requirements of the study and provided written informed consent. The sample primarily consisted of young adults, with an average age of 20.7 years (SD=3.19). Gender distribution was relatively balanced, but with respect to ethnicity the sample was skewed toward individuals identifying as Asian (51%). Full self-reported demographic details and scores on the Santa Barbara Sense of Direction Scale (SBSOD) [43], and the Spatial Anxiety Scale (SAS) [63] are presented in Table 2.

### 4.2 Environment

A hospital environment was chosen for this experiment because it is a scenario where effective navigation is crucial, affecting important outcomes such as patient care and operational efficiency. Hospitals are inherently complex, featuring intricate layouts, numerous intersections, and distinct landmarks, making them an ideal setting for evaluating spatial learning and wayfinding performance. By simulating these environments, we can closely mimic the challenges faced in various real-world contexts, allowing us to assess how VLTs perform under conditions that require accurate navigation and decision-making. This choice provides valuable insights that can inform the design of VR applications used in medical training, planning, and patient education, ensuring they are realistic, effective, and applicable to real-world needs.

**Figure 3:**
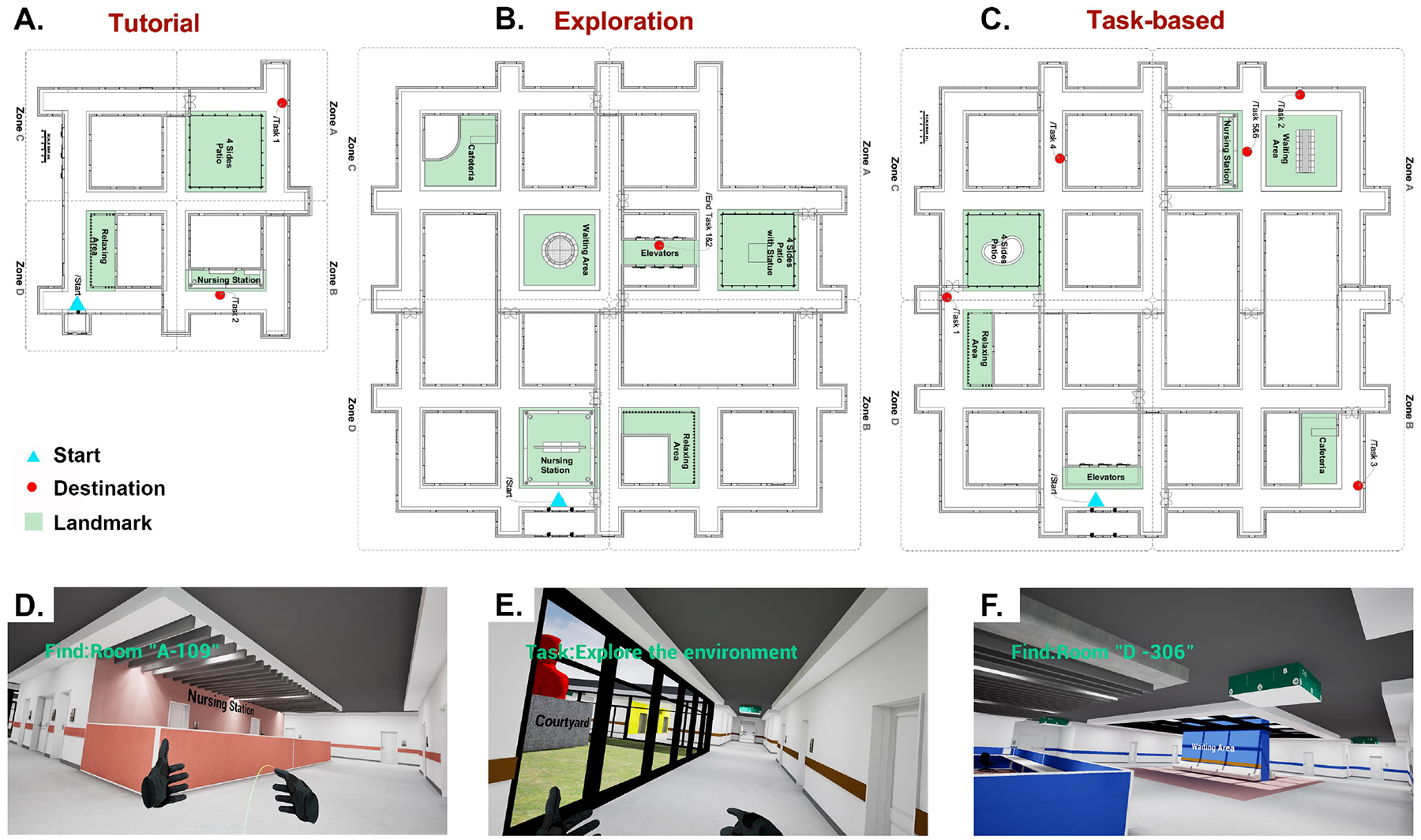
Plan views (A-C) and perspective views (D-F) of the virtual environments used in the study. On the plan views, landmarks are highlighted in green, and task start/end points are shown as red dots. Perspective views included a display of the task instructions that participants were completing.

The experiment’s design further supports this by including both an exploration phase and a task-based phase (Figure 3). The exploration phase offered a relaxed environment for spatial learning, allowing participants to familiarize themselves with the hospital layout at their own pace, which is essential for developing cognitive maps [24, 44]. In contrast, the task-based phase replicated real-world healthcare scenarios, where wayfinding tasks are performed under time constraints and specific goals, simulating the pressures and demands of actual navigation. This combination allowed us to evaluate how participants learn and apply spatial knowledge in both low-pressure and high-pressure settings, reflecting the realities of navigating complex hospital environments [108, 122].

The VR environment created for the study was designed to resemble a modern hospital interior, with dimensions of 40 m × 40 m × 3 m for the tutorial environment and 80 m × 80 m × 3 m for the exploration and task-based environments (Figure 3) Six key landmarks (Nursing Station, Relaxing Area, Four-Sided Patio, Elevators, Cafeteria, and Waiting Area) were clearly labeled with readable text and highlighted in distinct colors in both the exploration and task-based environments (Figures 2 and 3). Each environment was divided into four zones (A to D), with appropriate directional signage and room numbers (Figure 5) starting with the zone letter, such as room A109.

Modeling and UV mapping were primarily done using Autodesk 3ds Max, while Unreal Engine 5.4 (UE5) was used to program lighting, surface textures, and interactive features. Front-end interactions were developed using C++ and Blueprint visual scripting. Audio components were not included in the simulation. The simulation was packaged for standalone use and presented to participants using a Meta Quest 3 HMD, providing a resolution of 2064 × 2208 pixels per eye. The Meta Quest 3 can be adjusted for users with different interpupillary distances and offers a horizontal field of view of 110° and a refresh rate of 90 Hz. Participants experienced the virtual environment from a standing position. They could freely look around and observe various parts of the hospital space and navigate based on the assigned VLT. Participants’ behaviors and interactions were recorded in a customized UE5 log file associated with the participant ID. This included timestamps (in milliseconds) for their trajectories and each behavioral event (i.e., start/end task), along with the gaze direction. Responses to questionnaire instruments were collected directly within the VR and were included in the log file.

### 4.3 Procedure

**Figure 4:**
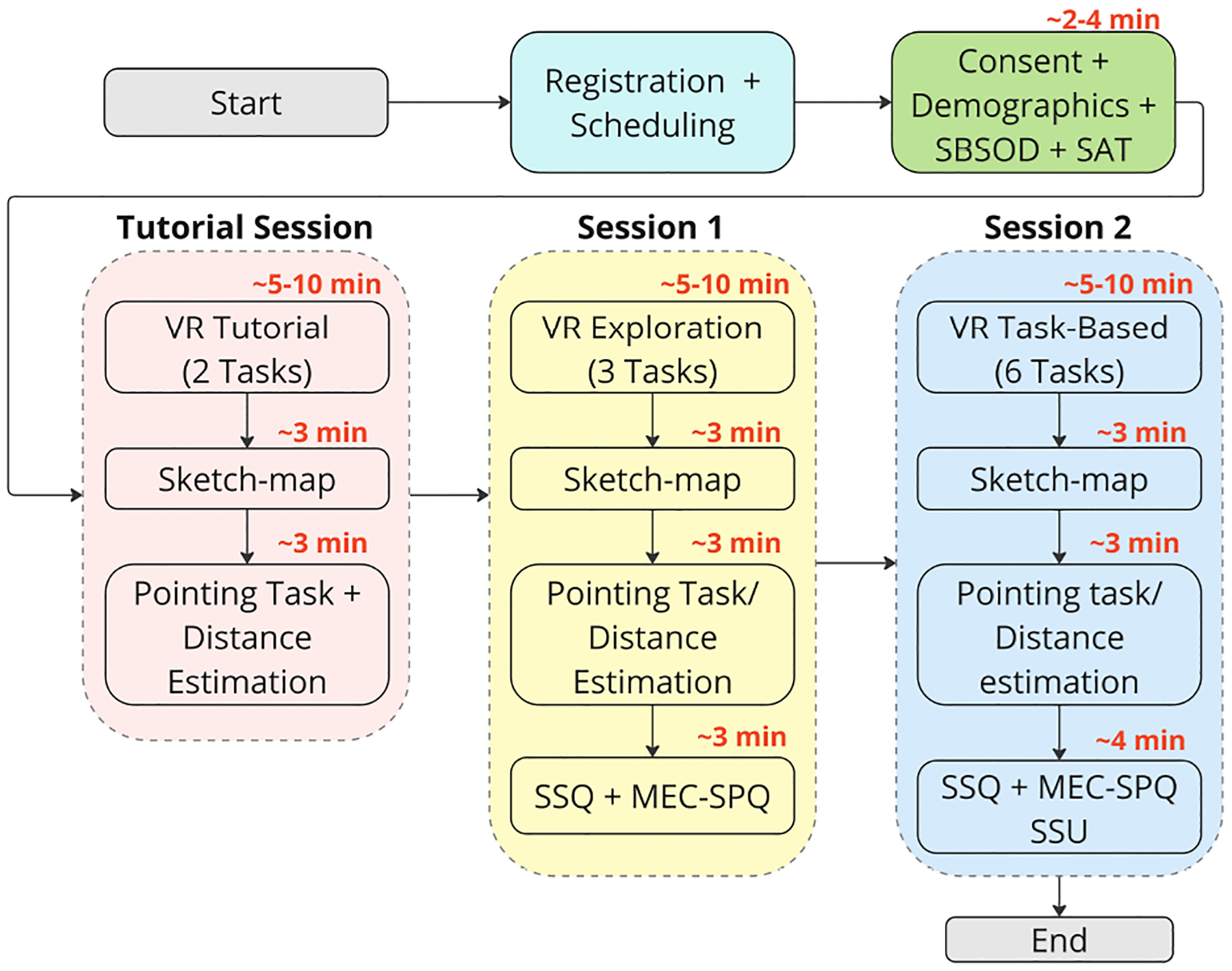
Overview of the experimental procedure for each participant, showing the sequence of recruitment, consent, demographics, tutorial exploration, task-based navigation, spatial assessments, sketch mapping, and surveys.

**Figure 5:**
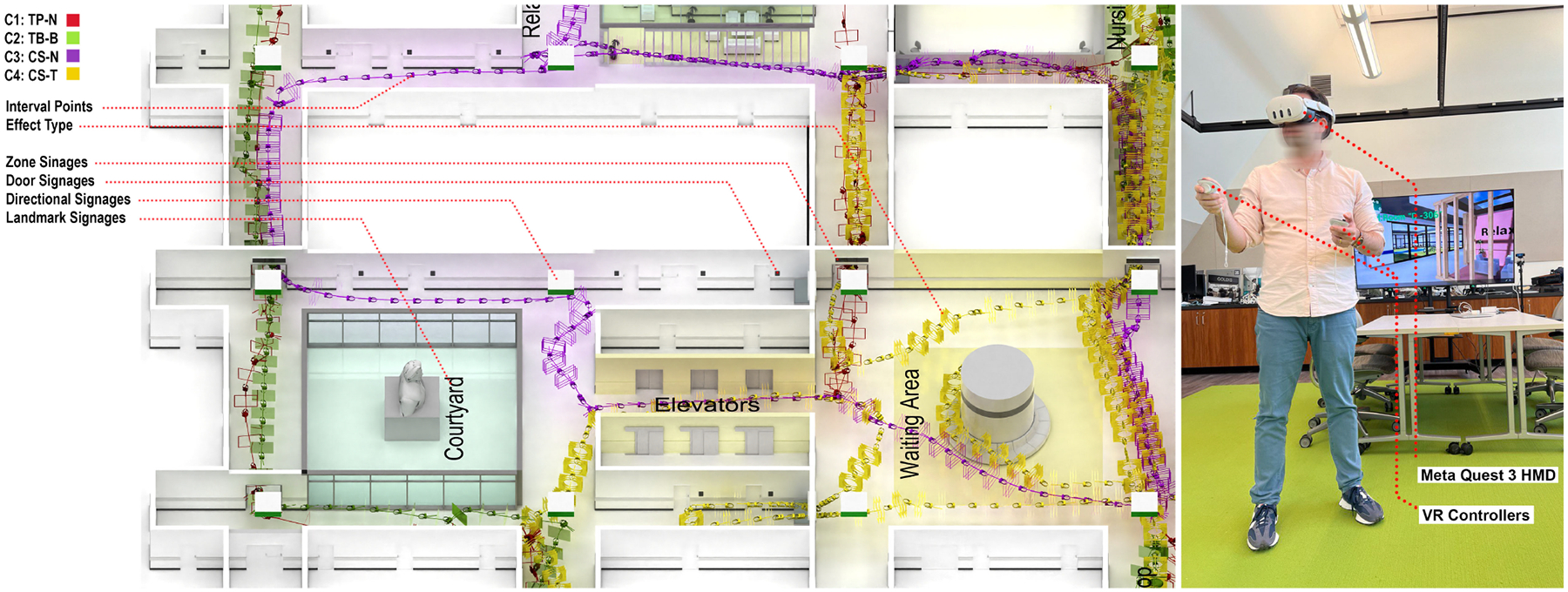
Trajectories of four specific participants as mapped on the exploration plan (left), and a participant during data collection in the laboratory (right).

The procedure of this study is illustrated in Figure 4, and the corresponding task details are shown in Table 3. Although the experiment included two independent variables, controller-based locomotion type (CS vs. TP) and viewpoint transition techniques (no effect vs. blinking or tunneling), the study was not conducted as a full factorial design because the effects of viewpoint transitions are not directly comparable between the TP and CS conditions. Following a between-subjects design, each participant was randomly assigned to one specific VLT condition, which was consistently used throughout the entire experiment. The real-world setting in which the VR-based study took place was a controlled laboratory environment in a university campus building.

Three environments were developed for this experiment (Figures 3 A, B, and C). The first environment, with relatively simple features, was intended for the tutorial session (Figure 3 D). The second and third environments, as detailed in Section 4.2, shared similar layout designs regarding the number of intersections, on-route environmental features, and overall navigation complexity, which allowed us to evaluate spatial learning and wayfinding effectiveness in comparable virtual environments.

To independently assess participants’ cognitive map development and navigation performance in relation to cybersickness and presence, and to avoid confounding factors such as the intrinsic differing speeds associated with VLTs, each participant completed two environments. Teleportation may allow for faster wayfinding compared to continuous locomotion, which could lead to unequal environmental learning time; therefore, this was controlled by using a fixed three-minute period. Therefore, spatial learning was evaluated by having participants spend three minutes exploring the second environment (Figure 3 E), followed by an assessment of their wayfinding performance in the third environment (Figure 3 F). Sessions were conducted individually and lasted approximately 45 minutes to one hour per participant.

Before the start of the session, participants were briefed on the study’s objectives and requirements and provided written informed consent. They also completed a brief survey regarding demographics and completed the Santa Barbara Sense of Direction Scale (SBSOD), and Spatial Anxiety Scale (SAS). Following this, participants were guided through the tutorial session, where they used the assigned VLT to explore the tutorial environment for a 5–10 minute period to become familiar with the Meta Quest 3 headset and navigational controls within the virtual environment by finding a room and a landmark (Tasks 1 and 2). They also received a tutorial on the sketch map task conducted on paper, as well as instructions on the pointing and distance estimation tasks on a tablet.

Next, participants went through the learning phase in the exploration environment, where they were asked to freely explore the second hospital environment for up to three minutes (Task 1), beginning at the designated point, with the objective of covering as much of the environment as possible while paying close attention to the landmarks and the overall layout of the environment. This setup is commonly employed in research on cognitive map development [122]. At the end of the exploration, they were tasked with completing a wayfinding performance trial within the same environment (Tasks 2 and 3), navigating from a starting point to locate a specific landmark (Elevator), and then repeating their steps to the same destination to assess their spatial knowledge [116]. Participants were then asked to complete a sketch map task by placing the landmarks on a simplified map [77, 78]. They also completed the pointing and distance estimation tasks related to previous landmarks and a self-report questionnaire to evaluate levels of cybersickness and presence, utilizing the Simulator Sickness Questionnaire [52] and MEC Spatial Presence Questionnaire [110].

After the exploration environment, participants were directed to the task-based environment, where they were tasked with four wayfinding trials in a continuous loop (Task 1 to 4), with each subsequent task starting where the previous one ended. The routes varied in length from 185 to 345 feet (56.3 to 105.1 meters) and incorporated different levels of complexity in regard to the number of intersections and on-route environmental features, allowing for a comprehensive evaluation of the VLTs’ impacts [101]. In the task-based environment, each participant was assigned to a starting task number and was instructed to find a particular room in the environment as quickly as possible for each trial. If participants were struggling or lost for more than ten minutes, or if they opted to abandon the task, then the researcher would promptly guide them to the end point. After completing the four wayfinding trials, participants were again tested with a navigation and route-retracting task (Task 5 and 6), followed by the sketch map and questionnaire, identical to the procedure from the exploration phase. Finally, they were asked to evaluate the usability of the VLT that they had experienced through the System Usability Scale [21].

### 4.4 Measures

We used two primary wayfinding performance measures: the time required for task completion and the distance traveled during each task. Time was automatically recorded from the moment participants began walking from the starting point until they reached the endpoint of each task. Participant trajectories on the floorplan were also captured using the built-in localization capabilities of the VR system to provide detailed movement data. In addition to these performance measures, spatial learning was assessed through a series of pointing tasks [45, 79] and distance estimations. These assessments were conducted after Tasks 2 and 3 in the exploration condition and after Tasks 1 through 6 in the task-based wayfinding condition, allowing us to evaluate participants’ cognitive map development and spatial knowledge acquisition.

**Figure 6:**
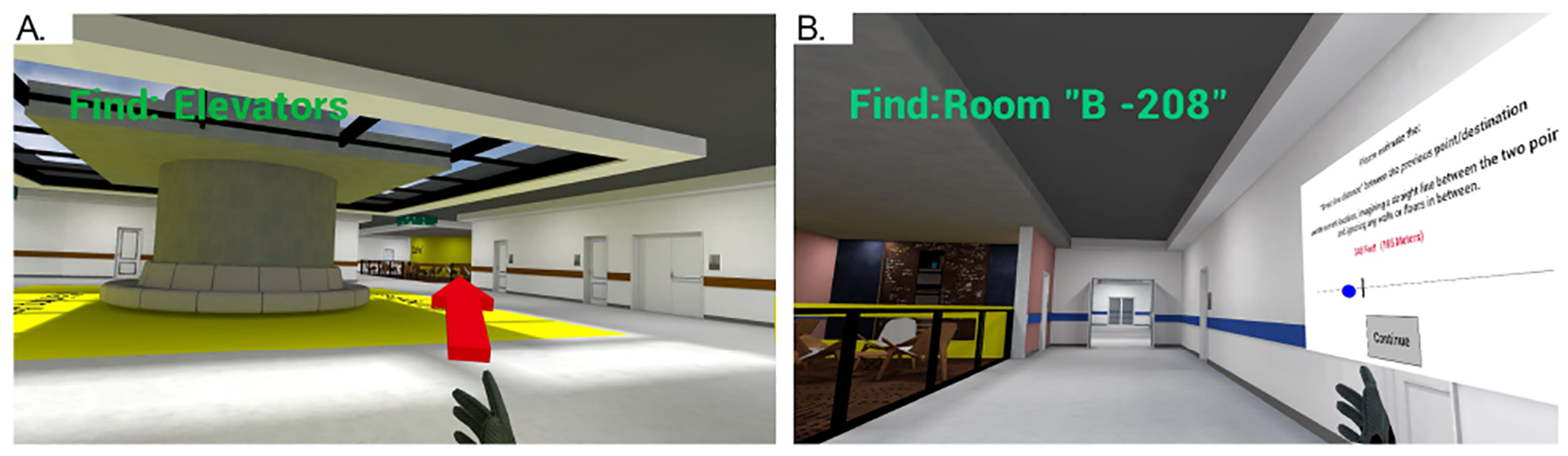
Screenshots from a participant’s headset: A. Pointing task in the Exploration level for Task 2; and B. distance estimation in the Task-based level for Task 3.

For the pointing task in VR, participants used the red arrow displayed on their virtual hand to indicate the direction of the previously instructed location and pressed the “A” button on the controller to confirm their response (see Figure 6). For the distance estimation task, participants were asked to estimate the straight-line distance between the two locations shown in the provided images [105]. Participants adjusted the slider on the screen to indicate the distance within a range of 0 to 1,000 feet (304.8 m). Distance estimation errors were determined as the discrepancy between the participant estimates and the actual straight-line distance.

After each environment, participants completed the sketch map followed by a survey (see Figure 4). For the sketch map task, participants were provided with a simplified map of the environment without landmarks on a letter-sized sheet of paper. The task required participants to recall and place all six landmarks on a map, including their names, positions, and sizes. The sketch maps were graded for accuracy across three criteria: landmark identification (6 possible points, 1 point for each correctly identified landmark, 0 for not identified), landmark placement (6 possible points, 1 point for fully accurate placement, 0.5 points if placed within one adjacent block, and 0 for incorrect placement or not identified), and spatial configuration (6 possible points, 1 point for fully accurate configuration, 0.5 points if within one adjacent block, and 0 for incorrect configuration or not identified) [77, 113].

The Simulator Sickness Questionnaire (SSQ) scores were calculated by summing (with weights) across the scale’s three components (“nausea,” “oculomotor,” and “disorientation”) and using a ranking of symptoms as none, slight, moderate, or severe [52], with a theoretical maximum of 235.62. The MEC Spatial Presence Questionnaire (MEC-SPQ) scores were averaged on a 5-point Likert scale across its two dimensions (“self-location” and “possible actions”) [110]. Scores on the System Usability Scale (SUS), a 10-item, 5-point Likert scale, were remapped to a 0–100 range [21].

### 4.5 Statistical Analysis

We used the R language for statistical analysis [88]. We first calculated descriptive results, then performed F-tests and estimated effect sizes *ω*^2^ with effectsize package [14] to examine the main effect of experimental conditions on the outcome measures, including learning phase behavior (duration, distance), wayfinding performance (duration, distance), spatial learning (pointing error, distance estimation error, sketch-map score), and user experience (cybersickness, spatial presence), adjusted for the fixed effect of task and random effect of participant.

For significant and marginally significant effects, we estimated differences and confidence intervals using the R emmeans package [65, 99] between condition pairs: TP-B vs. TP-N; CS-T vs. CS-N; and TP-N vs. CS-N. We reported test results with p-values less than 0.05 as significant and from 0.05 to 0.10 as marginally significant.

As secondary confirmatory analyses, we examined if Sense of Direction and Spatial Anxiety moderate the condition main effects on outcome measures. We also examined whether user experience measures predicted task performance and spatial learning outcomes. Linear mixed models were applied, adjusted for fixed effects of experimental condition and task, with participant as a random effect. Usability was excluded from the model due to its correlation with cybersickness (r = −0.41, t(264) = −7.23, p < 0.001). Because the two dimensions of spatial presence (self-location and possible actions) were highly correlated (r = 0.73), they were combined into a single composite measure. Cybersickness and (combined) spatial presence were both included in the model, as they are not significantly correlated (r = −0.07, t(265) = −1.15, p = 0.250).

## 5 Results

The overall descriptive and f-test results are summarized in Table 4. Overall, participants spent an average of 50.85 seconds (SD=38.03) and traveled an average distance of 108.10 m (SD=78.50) during each wayfinding task. They had an average pointing error of 71.71 degrees (SD=40.70), a distance estimation error (log-transformed ratio) of 0.59 (SD=0.43), and a sketch-map score of 8.74 (SD=4.28). They reported cybersickness levels of 38.96 (SD=38.49), and spatial presence of 3.34 (SD=0.99) on self-location, 2.81 (SD=0.92) on possible actions. After finishing both learning environments, participants reported an average SUS score of 66.44 (SD= 15.75), indicating a moderate to good system usability.

**Figure 7:**
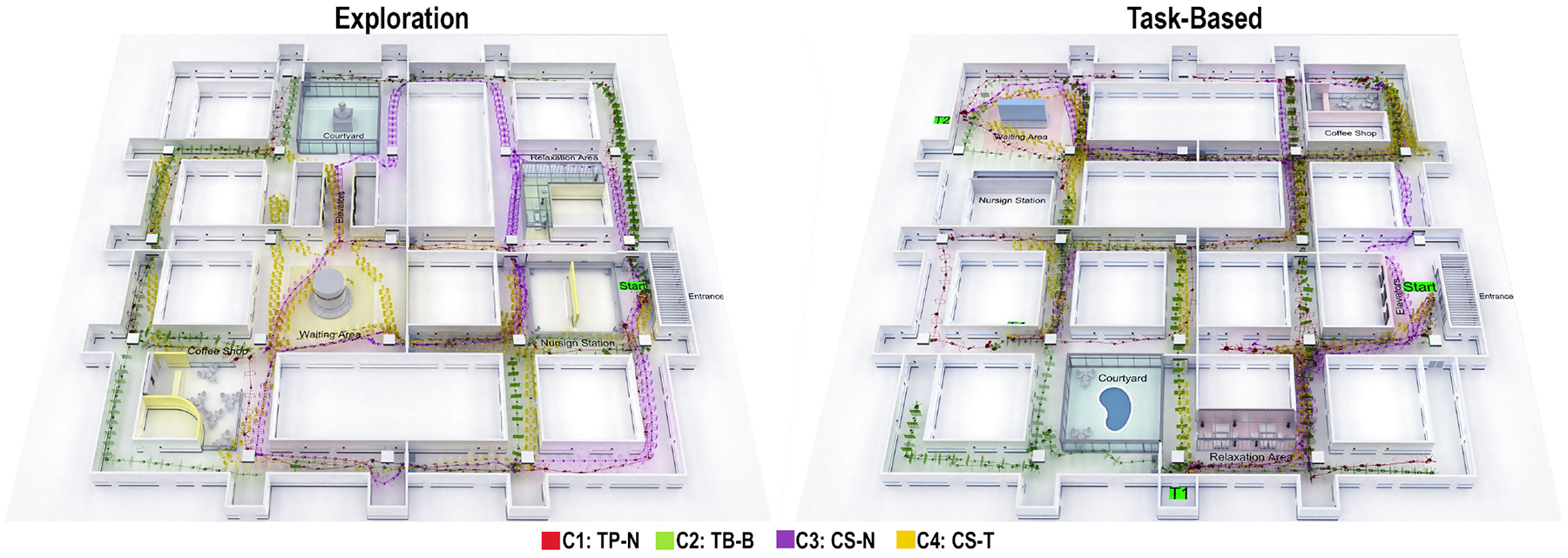
Participant trajectories in exploration (left) and task-based (right) environments across locomotion–transition conditions. Teleportation with blinking (TP-B) produced more direct routes, while continuous steering with tunneling (CS-T) showed smoother but longer paths, reflecting the trade-offs observed in the quantitative results.

Figure 7 shows a sample of participants’ trajectories in the exploration and task-based environments, illustrating differences in movement patterns under various VR conditions. For example, more erratic or fragmented paths and head direction were commonly observed in the teleportation condition with blinking transitions, suggesting lower spatial coherence or increased disorientation. In contrast, continuous steering combined with tunneling transitions often yielded smoother, more direct trajectories. These visual patterns provide qualitative support for the quantitative findings. Group means, standard deviations, and ANOVA results for the effect of each technological condition on the outcome metrics are provided in Table 4. The following sections discuss some of the most notable findings.

### 5.1 Wayfinding Performance

**Figure 8:**
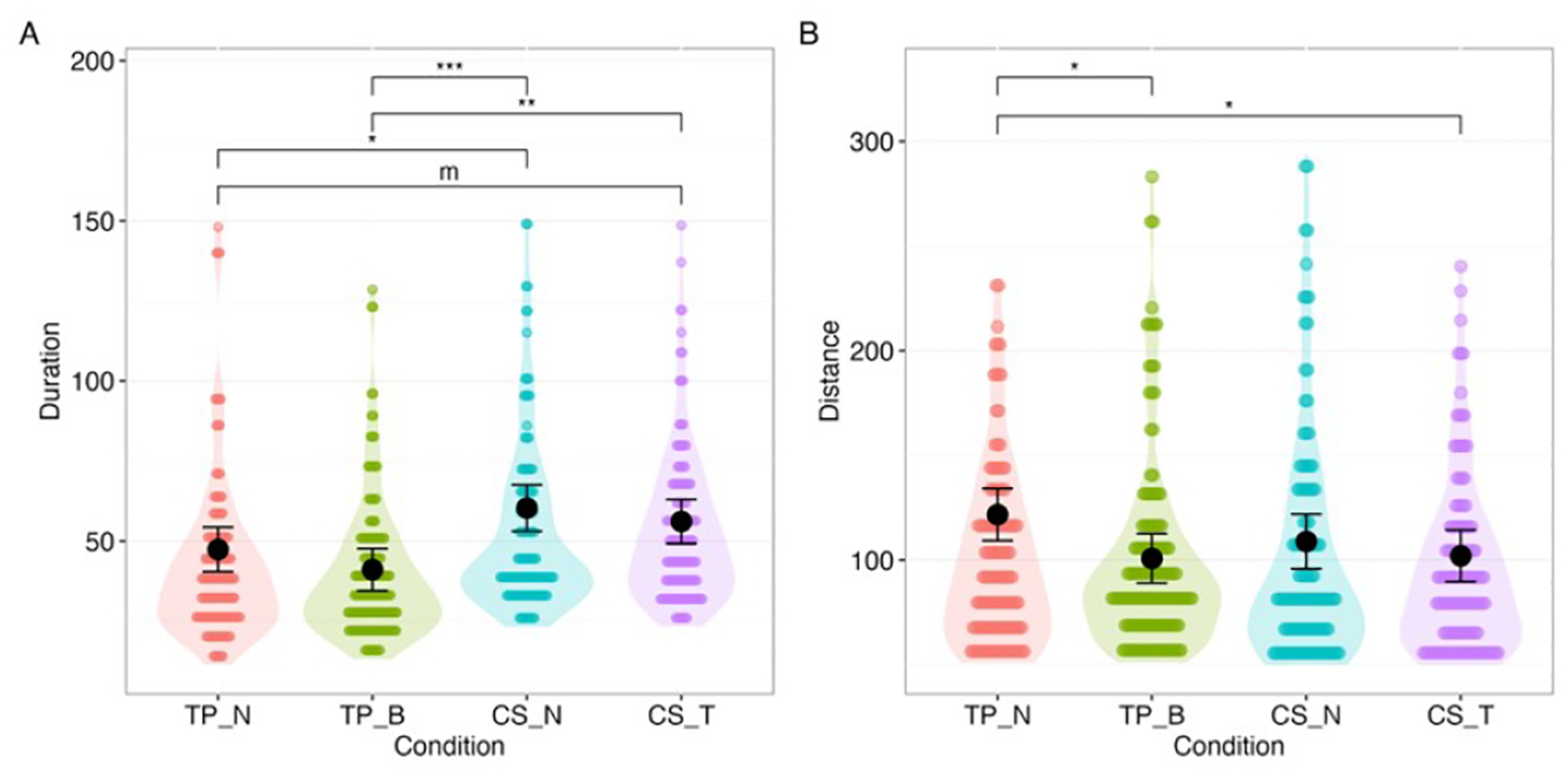
Distributions, estimated levels, and differences of: A. task duration and B. distance traveled. (For reference purposes, all pairwise results were plotted.) m: p<0.10, * p<0.05, **: p<0.01, ***: p<0.001

Task duration differed significantly across conditions, F(3, 138) = 6.21, p < .001, with a small effect size, *ω*^2^=0.10. Post hoc contrasts showed that the TP-N group completed tasks faster than the CS-N group (mean difference = −12.94 s, 95% CI [−22.95, −2.92]). Although the omnibus effect was significant, no other preplanned pairwise contrasts (TP-N vs. TP-B, CS-N vs. CS-T) reached significance (Figure 8A).

The effect of condition on distance traveled was marginal, F(3, 138)=2.38, p=0.072, with a small effect size, *ω*^2^=0.03. Within this trend, TP-N participants traveled significantly longer distances than TP-B (mean difference = −20.92 m, 95% CI [−38.06, −3.79]). No other preplanned contrasts (e.g., CS-N vs. TP-N, CS-N vs. CS-T) were significant (Figure 8B).

### 5.2 Cognitive Map Tasks

**Figure 9:**
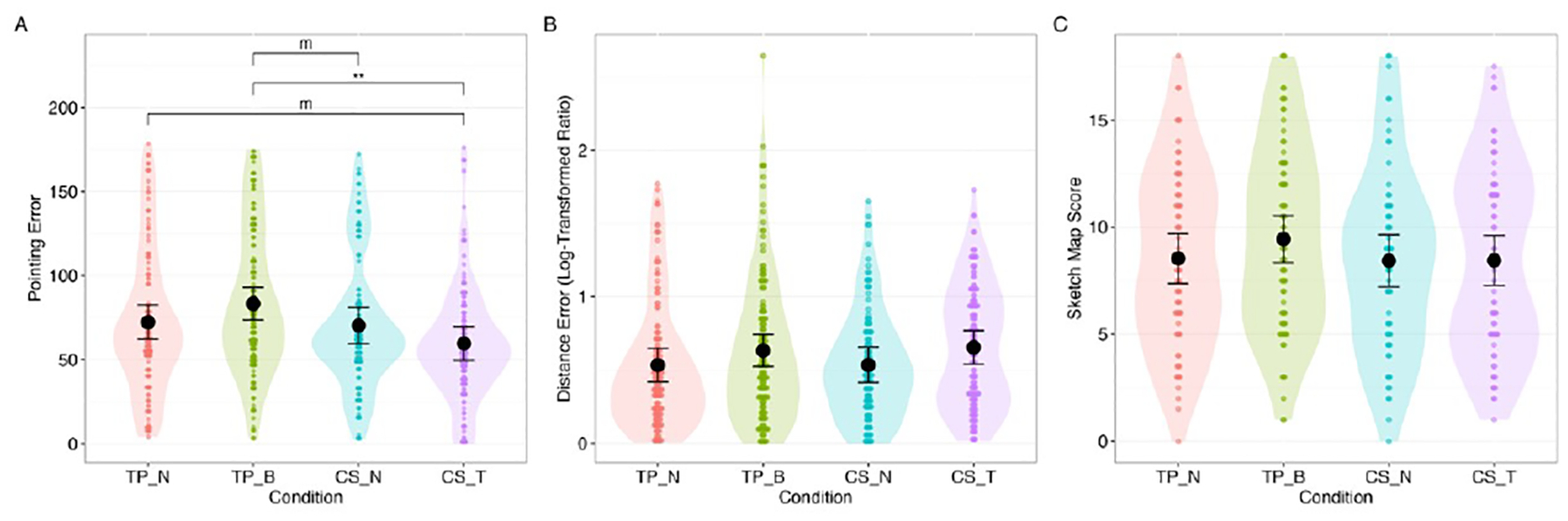
Distributions, estimated levels, and differences of: A. pointing error, B. distance estimation error, and C. sketch map score. (For reference purposes, all pairwise results were plotted.) m: p<0.10, * p<0.05, **: p<0.01, ***: p<0.001

Pointing error differed significantly across conditions, F(3, 139)=3.77, p=0.012, with a medium effect size, *ω*^2^=0.06. However, none of the preplanned pairwise contrasts (TP-B vs. TP-N, CS-T vs. CS-N, TP-N vs. CS-N) reached significance, suggesting that the overall effect was driven by differences between conditions not directly compared, with CS-T showing the lowest mean pointing error (Figure 9A).

There was no significant difference between experimental conditions in distance estimation error, F(3, 138)=1.20, p=0.311, *ω*^2^<0.01 (Figure 9B); or sketch map score, F(3, 137)=0.74, p=0.530, *ω*^2^<0.01 (Figure 9C).

### 5.3 User Experience

**Figure 10:**
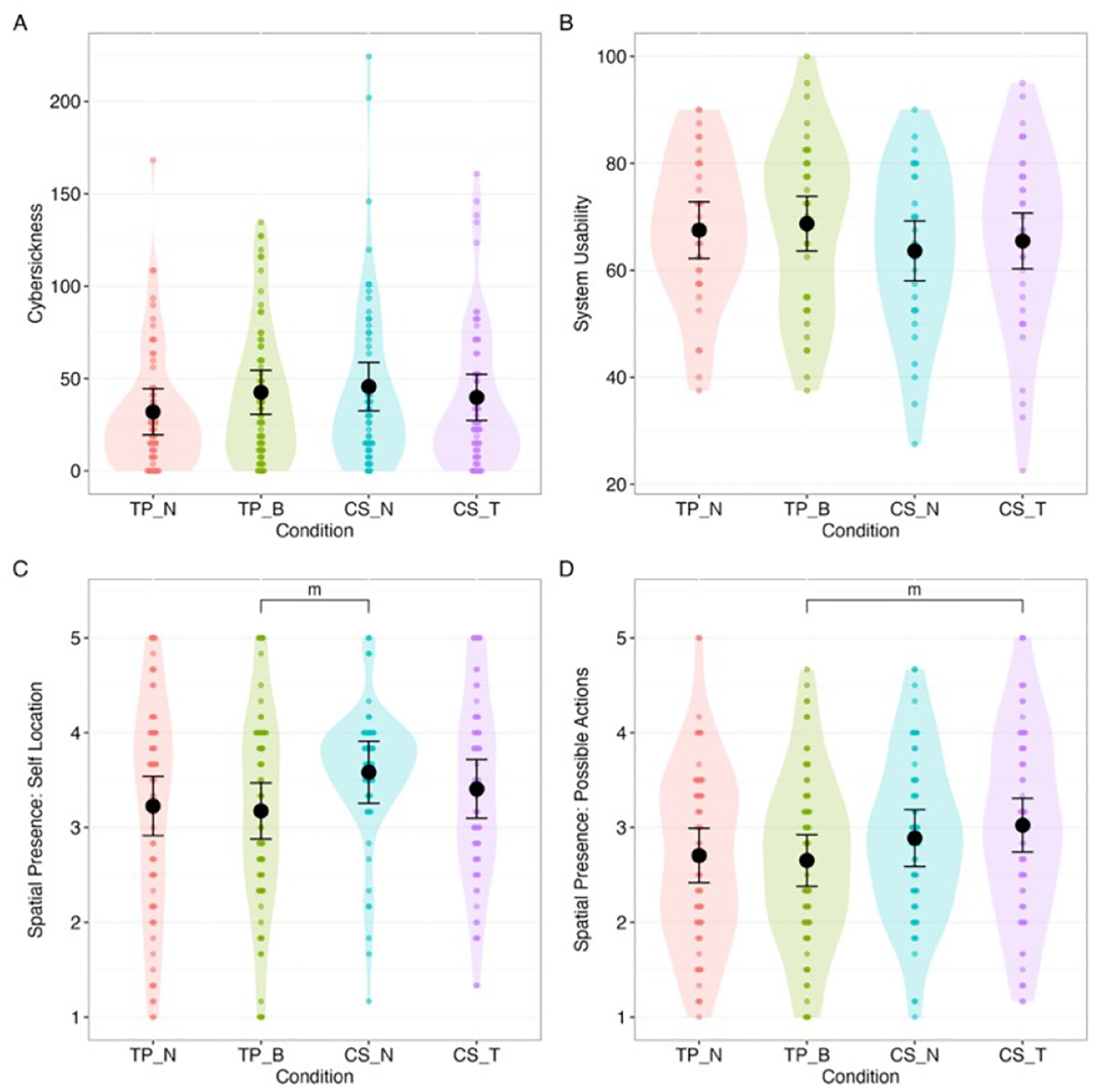
Distributions, estimated levels, and differences of: A. cybersickness, B. system usability, C. spatial presence: self-location, and D. spatial presence: possible action. (For reference purposes, all pairwise results were plotted.) m: p<0.10, * p<0.05, **: p<0.01, ***: p<0.001

There was no significant difference between experimental conditions in cybersickness, F(3, 136)=0.83, p=0.479, *ω*^2^<0.01 (Figure 10A); system usability score, F(3, 135)=0.68, p=0.566, *ω*^2^<0.01 (Figure 10B); self-location, F(3, 138)=1.35, p=0.260, *ω*^2^<0.01 (Figure 10C); or spatial presence: possible actions, F(3, 138)=1.44, p=0.232, *ω*^2^<0.01 (Figure 10D).

### 5.4 Effects of User Experience on Spatial Learning

In terms of effects of user experience on performance and spatial learning. Cybersickness significantly predicted pointing error (b = 0.14, SE = 0.06, t(224) = 2.24, p = 0.026) and sketch-map accuracy (b = −0.02, SE = 0.01, t(187) = −2.49, p = 0.014), with higher sickness associated with poorer spatial learning. No significant associations were found for task duration, distance traveled, or distance estimation. Spatial presence did not significantly predict any outcome measures (Table 5).

## 6 Discussion

This study set out to examine how interface-level choices in VR navigation, specifically the pairing of locomotion style and viewpoint transition, shape wayfinding performance, spatial learning, and user experience in complex indoor environments. Across a large between-subjects sample (N=142) and two complementary contexts (free exploration and time-pressured wayfinding), we observed clear performance advantages for teleportation with blinking (TP-B) alongside mixed, outcome-specific patterns for spatial cognition: continuous steering with FOV restriction (CS-T) tended to support finer-grained configurational knowledge (e.g., lower pointing error trends). Importantly, cybersickness and presence neither differed reliably by condition nor mediated condition effects, yet higher sickness modestly predicted poorer pointing accuracy and sketch-map scores. In what follows, we interpret these findings through the lens of the paper’s three contributions and position them relative to prior work.

### 6.1 A high-fidelity VR Framework for Wayfinding Research

By situating the experiment in realistic hospital layouts with controlled tutorial, exploration, and task-based phases, this framework addresses a gap in the literature that has largely favored outdoor, open, or abstract scenes with single-outcome evaluations (e.g., [18, 19, 114]). The two-stage design helped dissociate route efficiency from cognitive-map development, a confound in many studies where faster completion also reduces exposure time [89]. Our findings replicate and refine established trade-offs. Consistent with prior work [62], teleportation yielded faster completion, and adding a subtle blink transition further suppressed distracting optical flow while maintaining rapid target acquisition. While teleportation has often been linked to impaired metric updating due to absence of optic flow [22, 85], our TP-B results suggest that careful transition design can mitigate these costs by reducing disorientation while preserving efficiency. Conversely, continuous steering provided continuous motion cues that aided spatial updating and path integration [12, 13, 114] but extended task times. Unlike studies reporting strong condition effects on cybersickness across locomotion techniques (e.g., [36, 124]), our high-fidelity indoor setting and the use of a constant walking speed for Continuous Steering produced no overall main effects on SSQ. This aligns with Lin et al. [69], suggesting that in dense, task-rich indoor environments, modest parameterization can stabilize discomfort across techniques while still revealing performance–cognition dissociations.

### 6.2 An integrated Methodological approach

By combining behavioral logs, spatial-learning measures, and user-experience indices, the study captured multidimensional trade-offs that single-metric evaluations often overlook. For example, TP-B produced efficient routing but did not enhance configurational knowledge; CS-T yielded slower navigation but improved pointing accuracy. This aligns with findings that continuous optic flow aids spatial updating and cognitive mapping [28, 114], while abrupt viewpoint jumps fragment metric encoding unless mitigated by transition effects [16, 17]. The effect sizes of experimental conditions on navigation performance and spatial learning were small, suggesting a relatively high within-group variance possibly caused by individual differences.

Our results also extend prior evidence on cybersickness. Although average cybersickness did not differ by condition, individual differences mattered: participants with higher cybersickness performed worse on pointing and sketch-map tasks. This finding is consistent with prior reports that discomfort reduces attentional resources available for encoding landmarks [52, 61]. Importantly, presence ratings remained stable across locomotion styles, echoing results by Lin et al. [69] that well-parameterized VR systems can sustain immersion even under varying locomotion and transition techniques.

Results also suggest that individual differences in SBSOD and spatial anxiety did not moderate the comparative effects of locomotion techniques. In other words, the observed trade-offs between teleportation and continuous steering held consistently across participants with different self-reported spatial profiles (see Appendix A). While these points point to the robustness of the findings, future research with larger and more heterogeneous samples could explore whether individual traits influence locomotion preferences or effectiveness in more nuanced ways.

### 6.3 Foundations for VR Navigation Interface Design

Findings inform outcome-specific design recommendations. For applications requiring speed and directness (e.g., patient routing, gate-finding, or time-sensitive training), TP-B provides efficient navigation by minimizing visual distractions at the moment of transition. However, when configurational knowledge matters, techniques like CS-T, or other continuous steering variants that preserve optic flow, can balance efficiency and spatial updating [16]. Our results parallel those of Weissker et al. [114] and Barhorst-Cates et al. [12], who also found that continuous motion benefits spatial learning in dense, landmark-rich environments, though careful parameterization is required to avoid sickness [57, 59].

This adaptability ensures VR platforms can serve diverse user groups and task goals. Incorporating customizable locomotion settings, such as speed, rotational granularity, and transition style, empowers users to optimize for comfort, efficiency, or learning, aligning system capabilities with individual needs. More broadly, our study highlights the context sensitivity of locomotion effects: task demands, environmental density, and transition style interact to shape navigation strategies, explaining why teleportation sometimes impairs learning in sparse scenes but supports efficiency in complex, goal-driven contexts.

### 6.4 Implications for VLT Design for Wayfinding Research

The results of this study highlight the importance of aligning virtual locomotion technique (VLT) designs with the specific goals of the application, as different configurations demonstrated distinct trade-offs between task efficiency, spatial learning, user comfort, and immersion. Across both exploration and task-based wayfinding contexts, teleportation with a blinking transition (TP-B) consistently supported the fastest completion times but reduced exposure to environmental details, while continuous steering with field-of-view restriction (CS-T) fostered richer spatial understanding at the expense of slower navigation, with close levels of cybersickness. These findings underscore the necessity of flexible, customizable VLT interfaces that allow designers and users to balance competing demands based on context.

For tasks that prioritize speed and direct navigation, such as target-finding, airport gate simulations, or search-and-rescue training, TP-B appears to be the most suitable approach. The brief blinking transition likely reduces cognitive load during travel by filtering out irrelevant visual stimuli, allowing users to focus on destinations rather than environmental distractions. However, because TP-B reduces continuous optic flow, designers should be cautious when spatial learning or configurational understanding is critical. In these scenarios, basic teleportation without blinking (TP-N) offers a more balanced solution, preserving quick movement while improving awareness of landmarks and spatial relationships. TP-N is particularly relevant for time-sensitive environments, such as medical emergency response training, where users must move rapidly but still retain partial environmental knowledge.

In contrast, continuous steering without tunneling (CS-N) is well suited for applications emphasizing exploration and configurational learning, such as museum tours, architectural walkthroughs, or educational simulations. CS-N provides constant optic flow and uninterrupted peripheral visual information, which are essential for building accurate cognitive maps and improving path integration. However, designers must account for increased cybersickness risk, especially during extended sessions, by offering adjustable motion speeds, rotation sensitivity, and rest intervals.

The study also revealed that continuous steering with tunneling (CS-T) had the best performance in spatial learning as measured by pointing tasks, despite the persistent field-of-view restriction (for sketch-map accuracy and distance estimation, no significant differences were found among the conditions). Furthermore, the CS-T condition was not associated with a reduction in participants’ navigational performance compared to CS-N. These findings provisionally suggest that tunneling could be added to continuous steering with little, if any, negative impact, though further work is needed to confirm this conclusion. Related approaches, such as dynamic FOV restriction [36] should also be considered, as they may achieve a better balance by preserving peripheral awareness while still protecting user comfort.

For therapeutic and social applications, particularly those involving older adults or users sensitive to simulator sickness, simplicity and comfort should be prioritized. In these contexts, teleportation without subtle transition effects remains the most accessible solution due to its low physical exertion and reduced risk of disorientation. However, for applications aimed at navigational skill training or real-world transfer, continuous steering modes may still be preferable, provided they are paired with user-controlled adjustments for speed, rotation, and transition effects. This distinction is especially relevant for VR-based cognitive training and rehabilitation interventions, where locomotion choices must carefully balance comfort with opportunities for strengthening memory, orientation, and spatial awareness.

Ultimately, these findings argue for adaptive VLT design frameworks that allow users to personalize navigation settings based on their objectives, preferences, and comfort thresholds. By offering modular controls, including speed adjustments, snap-versus-smooth rotations, and optional transition effects, VR systems can better accommodate diverse user groups, tasks, and contexts. Such flexibility not only ensures that VR environments remain accessible and engaging but also enhances their utility both as experimental platforms for human–environment interaction research and as practical tools for designing interventions in healthcare, education, and rehabilitation.

## 7 Limitations And Future Work

While this study provides valuable insights into how locomotion and transition techniques shape navigation and spatial learning in VR, several limitations should be noted. First, the participant sample was skewed toward young, highly educated Asian adults, which may influence how individuals engage with VR technology and navigate complex spaces. Future work should recruit larger and more diverse populations to improve the generalizability of findings. Second, the duration of VR exposure was relatively short, limiting our ability to capture longer-term effects of locomotion techniques on spatial knowledge retention, wayfinding performance, and cybersickness. Extended longitudinal designs would help clarify whether the observed trade-offs persist over time. Third, the study did not address natural locomotion approaches such as physical walking or walking-in-place. Future studies could benefit by examining these modalities when spatial and technical conditions allow, enabling direct comparison with controller-based approaches.

Fourth, our study employed a partial factorial design, focusing on the most theoretically relevant locomotion–transition pairings. While this reduced the participants’ burden, it also limits the completeness of condition contrasts. Future research should test a fuller set of combinations to better disentangle locomotion and transition effects. Fifth, although the study used hospital-like layouts to ensure realism, the findings may not generalize across all domains of VR, such as gaming, education, or outdoor navigation. Finally, the study was conducted on Meta Quest 3 devices with a 90 Hz refresh rate and limited field of view. Although these parameters reflect common consumer-grade setups, future studies using higher-end headsets could clarify the role of display fidelity in shaping both performance and user comfort.

Future work could also integrate additional indicators such as eye-tracking, head-movement patterns, and micro-pauses to clarify how transitions alter attentional allocation. Combining such data with behavioral logs would strengthen cross-study reproducibility and provide a richer basis for linking interface parameters to cognitive outcomes.

## Figures and Tables

**Table 1:** Overview of prior research on VR locomotion and view transition techniques for spatial learning

Study	Technique(s)	Focus / Task	Key Findings	Relevance / Limitation
Bowman et al., 1997 [19]	Gaze-steering; pointing-steering; continuous vs jumping	Object-location & awareness	Jumping caused disorientation; continuous motion preserved awareness	Shows value of optic flow; tested in small/simple scenes
Bolte et al., 2011 [17]	Real walking; Jumper; Instant teleport	Object visiting + sketch-map	Best memory for walking; Jumper close; teleport worst	A hybrid jump is beneficial, but small N and simple scene
Weissker et al., 2018 [114]	Continuous steering vs. restricted jumping	City routes + pointing + SSQ	Jumping much faster & less sickness; similar updating overall	Individual differences; limited route complexity
Bhandari et al., 2018 [16]	Instant TP vs Dash (brief optical flow)	Landmark-free path integration	Dash improved path integration without adding sickness	Mobile VR, small N, simple scene, no continuous locomotion
Rahimi et al., 2018 [90]	Instant TP; animated; pulsed	Visual tracking during transitions	Animated best for spatial tracking; instant best for sickness	Applies to automated viewpoint shifts, not user locomotion
Adhikari, 2022 [5]	Lean-motion; controller; +HyperJump	Waypoint navigation + pointing	HyperJump faster; leaning improved orientation; slightly more frustration	Low sickness overall; small N; city-style constraints
Riecke et al., 2007 [93]	Landmarks vs. pure optic flow; platform rotation	Rotational updating tasks	Landmarks enabled automatic updating; optic flow alone is insufficient	Applies to rotational updating, not long-distance navigation
Klatzky et al., 1998 [56]	Walking; imagined; visual-only; rotation-mixed	Triangle-completion	Walking + real rotation best; visual-only and imagined failed	Shows need for vestibular + proprioceptive cues
Cherep et al., 2020 [28]	Walking; partial TP; discordant TP	Path-integration	Walking best; discordant TP worst; boundaries mitigated TP error	Highlights the role of environmental geometry
Lim et al., 2020 [67]	Partial vs discordant teleport	Object-location recall	Rotational cues improve survey knowledge	No walking or continuous motion included
Zielasko et al., 2022 [124]	4 discrete rotational methods	Rotation search + orientation	Selection-based fastest; no orientation difference	Rotation-only; no translation
Xu et al., 2017 [119]	Joystick; TP; walking-in-place	Object-location memory transfer	No difference between locomotion types	Task is simple; single-object recall
Paris et al., 2019 [85]	TP; grapple; skiing; magic carpet	Landmark-free path-integration	Continuous best for angular accuracy; no sickness or presence differences	No comparison to real walking; simple task
Buttussi & Chittaro, 2023 [23]	TP vs steering (VR vs tablets)	Distance & relational memory + retention	VR steering improved updating; TP had less nausea; mixed long-term effects	Results vary by knowledge type; some sample bias
Kim et al., 2024 [53]	Joystick; Teleport; RDW	Static vs dynamic VE	Joystick best in static; RDW best in dynamic; TP worst overall	RDW adds resets & sickness in return for accuracy
Kitson et al., 2017 [55]	Multiple interfaces vs joystick	Object search + UX	Joystick is most precise; motion-cueing is more engaging	Qualitative; no spatial-learning metrics
Rantala et al., 2021 [91]	Teleport; Slider; Grab	Corridor search	Continuous faster & less cognitive load than TP	Simple linear space; seated condition

**Table 2:** Summary of participant demographics, educational background, and baseline spatial characteristics across all experimental conditions, showing comparable group composition.

Demographics	TP-N (n=35)	TP-B (n=39)	CS-N (n=32)	CS-T (n=36)	Overall (n=142)
**Age**	20.97 (3.03)	20.50 (3.97)	20.09 (1.91)	21.17 (3.36)	20.70 (3.19)
**Gender**					
Female	20 (57%)	28 (72%)	20 (62%)	24 (67%)	92 (65%)
Male	12 (34%)	10 (26%)	12 (38%)	12 (33%)	46 (32%)
Non-binary	3 (9%)	1 (3%)	0 (0%)	0 (0%)	4 (3%)
**Ethnicity**					
No Primary Group	0 (0%)	0 (0%)	1 (3%)	0 (0%)	1 (1%)
White Caucasian	9 (26%)	13 (33%)	8 (25%)	7 (19%)	37 (26%)
Black/African American	3 (9%)	3 (8%)	4 (12%)	2 (6%)	12 (8%)
Asian	19 (54%)	19 (49%)	16 (50%)	18 (50%)	72 (51%)
American Indian/Alaska Native	1 (3%)	0 (0%)	0 (0%)	0 (0%)	1 (1%)
Native Hawaiian/Pacific Islander	0 (0%)	0 (0%)	0 (0%)	0 (0%)	0 (0%)
Multi-racial	1 (3%)	4 (10%)	3 (9%)	8 (22%)	16 (11%)
Prefer not to say	2 (6%)	0 (0%)	0 (0%)	1 (3%)	3 (2%)
**Education Level**					
Associate degree	8 (23%)	2 (5%)	5 (16%)	3 (8%)	18 (13%)
Bachelor’s degree	19 (54%)	25 (64%)	20 (62%)	23 (64%)	87 (61%)
Master’s degree	2 (6%)	2 (5%)	3 (9%)	4 (11%)	11 (8%)
Doctorate or professional	1 (3%)	1 (3%)	0 (0%)	0 (0%)	2 (1%)
**Sense of Direction ([1, 7])**	4.26 (1.05)	4.10 (1.01)	4.12 (1.01)	3.96 (0.84)	4.11 (0.98)
**Spatial Anxiety ([1, 5])**	2.65 (0.79)	2.44 (0.60)	2.70 (0.83)	2.91 (0.75)	2.67 (0.75)

*Notes:* Self-reported gender, ethnicity, and education level are shown as n and (%). Other measures are shown as mean and (SD).

**Table 3:** Summary of tasks completed within each environment, showing start and end locations and associated pointing/distance estimation assessments for all participants.

Task Number	Start Point	End Point	Pointing Task & Distance Estimation Location	Notes
**Tutorial**				
Task 1	Entrance	Room A109	To Entrance	
Task 2	Entrance	Nursing Station	To Entrance	
**Exploration**				
Task 1	Entrance	Anywhere in the Environment		
Task 2	Entrance	Elevator	To Entrance	Performance Task 1
Task 3	Entrance	Elevator	To Entrance	Performance Task 2
**Task-Based**				
Task 1	Entrance	Room D-306	To Entrance	
Task 2	Room D-306	Room A-101	Room D-306	
Task 3	Room A-101	Room B-208	Room A-101	
Task 4	Room B-208	Room C-110	Room B-208	
Task 5	Entrance	Nursing Station	To Entrance	Performance Task 1
Task 6	Entrance	Nursing Station	To Entrance	Performance Task 2

**Table 4:** Descriptive statistics, ANOVA results, and contrasts.

Measures	Descriptive				Main Effect	Contrast		
(Theoretical Range)	TP-N	TP-B	CS-N	CS-T	Condition	TP-B vs. TP-N	CS-T vs. CS-N	TP-N vs. CS-N
**Wayfinding Performance**								
Duration (seconds) (0, ∞)	47.49 (39.41)	40.92 (25.19)	60.36 (51.74)	56.51 (30.39)	*F***(3, 138)**=**6.21,** *p*<**0.001**, *ω*^2^=**0.10**	−6.29 (−15.82, 3.24)	−4.19 (−14.16, 5.78)	−12.94 (−22.95, −2.92)
Distance (meters) (0, ∞)	121.74 (112.59)	100.06 (49.22)	108.86 (79.04)	102.79 (60.21)	*F***(3, 138)**=**2.38,** *p*=**0.072**, *ω*^2^=**0.03**	−20.92 (−38.06, −3.79)	−6.85 (−24.79, 11.09)	12.84 (−5.17, 30.84)
**Cognitive Map Tasks**								
Pointing Error [0, 180]	72.35 (42.70)	83.36 (42.70)	70.41 (39.08)	59.42 (34.01)	*F***(3, 139)**=**3.77**, *p*=**0.012**, *ω*^2^=**0.06**	10.98 (−3.10, 25.06)	−10.71 (−25.42, 4.00)	2.02 (−12.78, 16.82)
Distance Estimation^a^ [0, 5.25]	0.53 (0.43)	0.63 (0.49)	0.54 (0.37)	0.66 (0.41)	*F* (3, 138)=1.20, *p*=0.311, *ω*^2^<0.01	0.10 (−0.06, 0.26)	0.12 (−0.05, 0.29)	0.00 (−0.17, 0.17)
Sketch Map [0, 18]	8.54 (4.14)	9.44 (4.34)	8.43 (4.31)	8.44 (4.32)	*F* (3, 137)=0.74, *p*=0.530, *ω*^2^<0.01	0.91 (−0.70, 2.51)	0.01 (−1.67, 1.70)	0.11 (−1.58, 1.79)
**User Experience**								
Cybersickness [0, 235.62]	32.12 (32.46)	41.24 (36.36)	44.76 (45.90)	38.16 (38.77)	*F* (3, 136)=0.83, *p*=0.479, *ω*^2^<0.01	10.53 (−6.73, 27.79)	−5.83 (−23.94, 12.28)	−13.62 (−31.74, 4.50)
Self-Location (MEC) [1, 5]	3.23 (1.13)	3.18 (1.04)	3.58 (0.72)	3.41 (0.96)	*F* (3, 138)=1.35, *p*=0.260, ω^2^<0.01	−0.05 (−0.48, 0.38)	−0.18 (−0.63, 0.27)	−0.36 (−0.81, 0.10)
Possible Actions (MEC) [1, 5]	2.70 (0.92)	2.65 (0.89)	2.89 (0.85)	3.03 (0.98)	*F* (3, 138)=1.44, *p*=0.232, *ω*^2^<0.01	−0.05 (−0.45, 0.34)	0.14 (−0.28, 0.55)	−0.18 (−0.6, 0.23)
System Usability [0, 100]	67.50 (13.84)	68.72 (16.21)	63.63 (15.56)	65.49 (17.30)	*F* (3, 135)=0.68, *p*=0.566, *ω*^2^ <0.01	1.22 (−6.15, 8.58)	1.86 (−5.8, 9.51)	3.87 (−3.84, 11.58)

a Distance Estimation is a log-transformed ratio.

*Note:* Mean (SD) for descriptive, estimated difference (95% CI) for contrasts. As this is not a 2 × 2 design, we only compared relevant pairs (TP-B vs. TP-N, CS-T vs. CS-N, and TP-N vs. CS-N).

**Table 5:** Effects of User Experience on Spatial Learning

Predictor	Duration	Distance	Pointing	Distance Estimation	Sketch Map Score
**Cybersickness**	*b*=0.03 (SE=0.05) *t*(165)=0.57, *p*=0.569	*b*=0.05 (SE=0.09) *t*(165)=0.552, *p*=0.582	*b*=**0.14 (SE=0.06)** *t* **(225)=2.242,** *p*=**0.026**	*b*=0.00 (SE=0.00) *t*(245)=−0.904, *p*=0.367	*b*=−0.02 (SE=0.01) *t*(187)=−2.492, *p*=0.014
**Spatial Presence**	*b*=−0.5 (SE=1.05) *t*(169)=−0.477, *p*=0.634	*b*=−2.58 (SE=1.92) *t* (169)=−1.346, *p*=0.180	*b*=**1.07 (SE=1.36)** *t* **(238)**=**0.786,** *p*=**0.433**	*b*=−0.01 (SE=0.02) *t*(264)=−0.522, *p*=0.602	*b*=0.01 (SE=0.16) *t*(191)=0.061, *p*=0.951

## CCS Concepts

- Human-centered computing → Mixed / augmented reality.

## A Moderation Effects of Sense of Direction and Spatial Anxiety

There was no interaction effect of Sense of Direction by experimental condition, or Spatial Anxiety by experiential condition, on any of the outcome measures (Table 6).

**Table 6:** Moderation Effects of Sense of Direction and Spatial Anxiety

Measures	Sense of Direction by Condition Effect	Spatial Anxiety by Condition Effect
**Wayfinding Performance**		
Duration	*F* (3, 133) = 1.38, *p* = 0.253, *ω*^2^<0.01	*F* (3, 134) = 1.32, *p* = 0.270, *ω*^2^<0.01
Distance	*F* (3, 133) = 0.84, *p* = 0.473, *ω*^2^<0.01	*F* (3, 134) = 0.61, *p* = 0.609, *ω*^2^<0.01
**Cognitive Map Tasks**		
Pointing Error	*F* (3, 134) = 0.56, *p* = 0.642, *ω*^2^<0.01	*F* (3, 135) = 0.19, *p* = 0.903, *ω*^2^<0.01
Distance Estimation	*F* (3, 134) = 0.60, *p* = 0.619, *ω*^2^<0.01	*F* (3, 134) = 0.48, *p* = 0.700, *ω*^2^<0.01
Sketch Map	*F* (3, 133) = 1.03, *p* = 0.381, *ω*^2^<0.01	*F* (3, 133) = 1.15, *p* = 0.333, *ω*^2^<0.01
**User Experience**		
Cybersickness	*F* (3, 131) = 0.86, *p* = 0.463, *ω*^2^<0.01	*F* (3, 131) = 0.88, *p* = 0.454, *ω*^2^<0.01
Self-Location (MEC)	*F* (3, 134) = 0.32, *p* = 0.813, *ω*^2^<0.01	*F* (3, 134) = 1.02, *p* = 0.387, *ω*^2^<0.01
Possible Actions (MEC)	*F* (3, 135) = 0.71, *p* = 0.546, *ω*^2^<0.01	*F* (3, 134) = 1.06, *p* = 0.369, *ω*^2^<0.01
System Usability	*F* (3, 131) = 0.13, *p* = 0.942, *ω*^2^<0.01	*F* (3, 131) = 0.64, *p* = 0.588, *ω*^2^<0.01

*Note:* The two moderators were tested separately.
